# The Hippo Pathway Effectors YAP/TAZ Are Essential for Mineralized Tissue Homeostasis in the Alveolar Bone/Periodontal Complex

**DOI:** 10.3390/jdb10010014

**Published:** 2022-03-01

**Authors:** Mirali Pandya, Gokul Gopinathan, Connie Tillberg, Jun Wang, Xianghong Luan, Thomas G. H. Diekwisch

**Affiliations:** 1 Center for Craniofacial Research and Diagnosis, Texas A&M College of Dentistry, 3302 Gaston Avenue, Dallas, TX 75252, USA; pandya@tamu.edu (M.P.); gopinath@tamu.edu (G.G.); ctillberg@tamu.edu (C.T.); xianghong.luan@tamu.edu (X.L.); 2 Department of Pediatrics, McGovern Medical School, The University of Texas Health Science Center at Houston, 6431 Fannin Street, Houston, TX 77030, USA; jun.wang@uth.tmc.edu

**Keywords:** Hippo pathway, YAP1, agrin, unopposed molar

## Abstract

YAP and TAZ are essential transcriptional co-activators and downstream effectors of the Hippo pathway, regulating cell proliferation, organ growth, and tissue homeostasis. To ask how the Hippo pathway affects mineralized tissue homeostasis in a tissue that is highly reliant on a tight homeostatic control of mineralized deposition and resorption, we determined the effects of YAP/TAZ dysregulation on the periodontal tissues alveolar bone, root cementum, and periodontal ligament. Loss of YAP/TAZ was associated with a reduction of mineralized tissue density in cellular cementum and alveolar bone, a downregulation in collagen I, alkaline phosphatase, and RUNX2 gene expression, an increase in the resorption markers TRAP and cathepsin K, and elevated numbers of TRAP-stained osteoclasts. Cyclic strain applied to periodontal ligament cells resulted in YAP nuclear localization, an effect that was abolished after blocking YAP. The rescue of YAP signaling with the heparan sulfate proteoglycan agrin resulted in a return of the nuclear YAP signal. Illustrating the key role of YAP on mineralization gene expression, the YAP inhibition-related downregulation of mineralization-associated genes was reversed by the extracellular matrix YAP activator agrin. Application of the unopposed mouse molar model to transform the periodontal ligament into an unloaded state and facilitate the distal drift of teeth resulted in an overall increase in mineralization-associated gene expression, an effect that was 10–20% diminished in Wnt1Cre/YAP/TAZ mutant mice. The unloaded state of the unopposed molar model in Wnt1Cre/YAP/TAZ mutant mice also caused a significant three-fold increase in osteoclast numbers, a substantial increase in bone/cementum resorption, pronounced periodontal ligament hyalinization, and thickened periodontal fiber bundles. Together, these data demonstrated that YAP/TAZ signaling is essential for the microarchitectural integrity of the periodontium by regulating mineralization gene expression and preventing excessive resorption during bodily movement of the dentoalveolar complex.

## 1. Introduction

The dentoalveolar complex is a unique functional and structural entity susceptible to relative displacement in relationship to the underlying jawbone [1,2]. The displacement of the entire dentoalveolar complex in mesial (humans) or distal direction (rodents) is termed drift [1]. Alternatively, the process of teeth erupting beyond the plane of occlusion within their sockets is called supereruption [1,3]. Under physiological conditions, the intricate microarchitecture of the dentoalveolar organ is little affected by tooth drift, resulting in the bodily displacement of the entire dentoalveolar organ while maintaining the integrity of the alveolar bone sockets, the thickness of the periodontal ligament, and the surface covering of the tooth roots (root cementum) [4]. The movement of teeth and their surrounding bony attachment apparatus is important for the life of organisms: a disrupted tooth row allows for food particles to be retained and cause caries, reduces the chewing and cutting efficacy of the dentition, diminishes individual esthetics, and affects physiological speech and/or vocalization.

The bodily movement of the dentoalveolar organ involves continuous modeling of the alveolar socket in the direction of the movement while maintaining the thickness of the periodontal ligament and the integrity of the dental root cementum [4]. In previous studies we demonstrated that the matricellular protein osteopontin plays a significant role in facilitating tooth drift by recruiting unloading-induced osteoclasts and modulating RANKL expression [2]. While the synchronous timing of osteoclast activity and the coordinated deposition of new bone to facilitate the drift of multirooted tooth rows is an extraordinary achievement all on its own, it is noteworthy that the basic microanatomy of the dentoalveolar apparatus consisting of tooth roots anchored within thin alveolar walls by means of a nonmineralized periodontal ligament remains unchanged regardless of the prevalent osteoclast activity involved [2]. It is equally remarkable that in physiological jaws, the integrity of the periodontal apparatus during dental drift remains intact under the extreme mechanical forces that are applied on the dentoalveolar organ during the occlusion of jaws.

Humans chew approximately 3000 times a day [5], resulting in forces up to 500 to 700 N applied to the dental attachment apparatus approximately twice every minute. Within the periodontal apparatus, occlusal forces affect a range of cells, including cementoblasts and cementocytes, periodontal ligament fibroblasts, and alveolar bone osteocytes, through compression and tension of the periodontal extracellular matrices (ECM) of cementum, periodontal ligament, and alveolar bone. ECM signals are then transmitted onto mechanosensitive molecules on the plasma membrane, especially focal adhesion protein complexes, which are comprised of integrins, vinculin, talin, and focal adhesion kinase [6,7,8]. Mechanoreceptors on the plasma membrane and cytoskeletal deformation in turn activate mechanotransduction pathways, including the Rho/ROCK pathway [9]. Mechanical information also causes modifications of several unique proteins, including β-catenin, paxillin, and the Hippo pathway members YAP and TAZ, to shuttle from the plasma membrane or the cytoskeleton to the nucleus [10,11]. ECM stimuli prompt YAP and TAZ to shuttle inside the nucleus, where they interact with transcription factors to affect cell–matrix interactions, ECM composition, and cytoskeletal integrity [12,13,14,15]. In this feed-forward control system, YAP is controlled by the ECM while in turn altering its composition [8,16]. One example for the interplay between the extracellular matrix and YAP signaling is the effect of the ECM proteoglycan agrin on YAP. Agrin transduces matrix rigidity signals and antagonizes focal adhesion assembly of core Hippo components by negating the functions of the Hippo pathway members Merlin and LATS1/2, thus serving as an ECM mechanotransduction signal [17].

The periodontal ligament is not mineralized, primarily due to the effect of Wnt signaling inhibitors [5]. In contrast, alveolar bone and cementum consist of mineralized extracellular matrices, mostly collagen as the organic matrix protein and hydroxyapatite as the inorganic matrix mineral. Matrix mineralization is promoted by the osteoblast transcription factor RUNX2, the enzyme alkaline phosphatase, and other mineralization proteins such as osteocalcin (OCN). While the multiple effects of the Hippo–YAP signaling pathway in osteoblast differentiation are still controversial [18], it has been demonstrated that YAP promotes osteogenesis and suppresses adipogenic differentiation by regulating β-catenin signaling [19]. YAP has also been associated with the differentiation and mineralization of cementoblasts [20].

In the present study we sought to determine the effect of the loss of Hippo signaling on the anatomy of the tooth row and on mineralized tissue homeostasis of the dentoalveolar apparatus. We asked how the loss of YAP affects mineralization gene expression in periodontal ligament cells exposed to cyclic strain and whether the extracellular matrix proteoglycan agrin would rescue the effect of the loss of YAP on mineralization gene expression. To verify whether Hippo effector dysregulation under mechanical loading conditions and in unloaded controls affects periodontal mineral homeostasis we examined dentoalveolar complex mineralization and gene expression in an unloading model. Together, the results from this study provided evidence for the intricate relationship between Hippo signaling and the integrity of the periodontal region under mechanical stress.

## 2. Methods

### 2.1. Animal Model

All animal procedures were approved by and followed the guidelines provided by the Texas A&M College of Dentistry IACUC committee and ARRIVE. Wwtr1^tm1Hmc^ Yap1^tm1Hmc^/WranJ mice were obtained from the Jackson lab (JAX stock #030532) [21] and crossed with Wnt1-Cre mice, also from the Jackson lab. These mice were bred according to the Texas A&M IACUC protocol and then genotyped following the genotyping protocol associated with each strain developed by the Jackson lab. Taz knockouts with Yap heterozygous genotypes positive for Wnt1-Cre were considered as experimental knockout (K/O) models (hereafter mentioned as Wnt1Cre/YAP/TAZ) while C57 BL6/J mice were used as true control wild type (WT). The Wnt1-Cre mice successfully crossed with the TAZ/YAP double-floxed mice (Wnt1^cre^ × TAZ^fl^YAP^fl^) were used for further studies. All mice were sacrificed as per the guidelines of the Texas A&M College of Dentistry IACUC committee.

### 2.2. Unopposed Molar Model

Study Design: Six adult WT and six adult K/O mice (8 weeks old) were used in the study. All right maxillary molar teeth from all twelve mice were extracted under general anesthesia and the mice were sacrificed 10 days after the extraction of the molars. The 10-day interval was optimum to capture the early events of tissue remodeling following the establishment of an unloaded condition [2]. Mandibles from the unopposed right side were collected for the experimental group while the contralateral sides served as controls. Half of the samples were frozen immediately upon sacrifice for molecular analysis while the other half were fixed in 10% formalin for paraffin and ground sections.

### 2.3. Isolation of Primary Periodontal Ligament Cells

Primary human periodontal ligament cells (PDL) were isolated from healthy first premolars obtained from patients undergoing tooth extractions for orthodontic indications following a previously established method [22]. The study protocol to obtain teeth and generate cells was in accordance with Texas A&M IRB committee guidelines. Written informed consent was obtained from all patients.

Briefly, periodontal tissues attached to the apical half of the root surface were collected in 1X DMEM (Sigma-Aldrich, St. Louis, MO, USA) using a sterile scalpel. The tissues were further cut into smaller pieces and digested with collagenase/dispase (Roche, Basel, Switzerland) for 30 min at 37 °C. The digested tissues were collected by centrifugation, incubated in DMEM supplemented with 10% fetal bovine serum, 100 U/mL penicillin, and 100 μg/mL streptomycin at 37 °C, 5% CO_2_ in a humidified incubator to allow individual cells to migrate out of the tissues. Media was refreshed every 48 h and, once confluent, the PDL cells were subcultured. At least three independent primary PDL cell populations were used at an early passage (P2–P4) for all experiments.

### 2.4. Radiographic Imaging

Hemimandibles from the WT, K/O, and the unopposed K/O model were analyzed using a Faxitron MX-20 specimen radiography system (Faxitron X-ray Corp, Lincolnshire, IL, USA) at 20 kV for 20 s.

### 2.5. Histology

For the present study, C57 BL6/J mice were used as true control wild type (WT) and the mandibles from both WT and K/O models were fixed in 10% buffered formalin for 1 week. For paraffin sections, the samples were decalcified for 4 days with 4.5% EDTA in a precision pulsed microwave oven (Electron Microscopy Sciences, Hatfield, PA, USA). Following decalcification, specimens were dehydrated, embedded in paraffin, and cut into 5 μm thin sagittal sections along the long axis of the molar teeth. To detect the presence of osteoclasts in the WT and K/O models, paraffin sections were stained for TRAP using the Sigma acid phosphatase detection kit 386A (Sigma-Aldrich) following the manufacturer’s instructions.

For the ground sections, all mandibles were subjected to dehydration via a series of different gradients of alcohol as per the EXAKT company standard protocol for preparation of tissues for ground sections. Once the samples reached the 100% light cure technovit stage (Technovit 7200, EXAKT Technologies Inc., Oklahoma City, OK, USA), they were polymerized via UV light exposure and embedded. The samples were grossly sectioned using a diamond bandsaw (EXAKT 300 CP) and further ground and polished to produce 45 μm thin sections. These sections were stained with von Kossa’s stain, which is a mineralized tissue marker. For von Kossa’s procedure, tissue sections were treated with 5% silver nitrate solution as previously described [23] and then stained for half an hour. After staining, the samples were counterstained with nuclear fast red stain and were analyzed using Leica light microscope (Leica, Frankfurt, Germany).

Trichrome staining of tissue sections was essentially performed as described by the manufacturer (Abcam, Waltham, MA, USA). Briefly, deparaffinized and rehydrated sections were placed in Bouin’s fixative at 56 °C for 1 h. After thorough washing, the sections were stained in Weigert’s iron hematoxylin for 10 min followed by Gomori’s trichrome stain for 15 min [24]. After rinsing with 1% acetic acid, sections were dehydrated and cleared through ethanol series and xylene and mounted with xylene-based mounting medium (Permount, Fisher Scientific, Waltham, MA, USA).

### 2.6. Application of Mechanical Stress on Cells

PDL cells (2 × 10^5^ cells/well) were seeded on amino or collagen I bonded flexible-bottomed 6-well culture plates for 24 h before treatment. For the agrin treatment, recombinant agrin (R&D Systems, Minneapolis, MN, USA) was added to the plates (diluted in 1X PBS to 2 μg/mL) and incubated at 37 °C for 2 h before seeding cells. Media was again supplemented with agrin (200 ng/mL) before mechanical stimulation of cells. Cells were subjected to mechanical stress by application of equibiaxial cyclic strain forces (15% strain, 0.1 Hz) in the FX-6000T tension system (Flexcell International, Burlington, NC, USA) as described before [25]. These parameters were based on previous studies of cellular mechanisms and differentiation gene markers induced by mechanical stress in PDL cells [25]. Cells were processed for total RNA extraction and immunofluorescence microscopy after 48 h of mechanical strain. PDL cells grown in plates not subjected to mechanical strain served as controls.

### 2.7. siRNA Mediated Knockdown of YAP

PDL cells were seeded in 6-well flexible-bottomed culture plates (2 × 10^5^ cells/well) prior to siRNA treatment. siRNA specifically targeting human YAP1 transcripts or nontargeting siRNA were obtained from Dharmacon (SMART Pool siRNA, Dharmacon, Lafayette, CO, USA) and used at a concentration of 60 nM using the DharmaFect 1 transfection reagent (Dharmacon). After siRNA treatment, cells were first incubated for 24 h before being subjected to mechanical strain for a total of 48 h.

### 2.8. Immunofluorescence Microscopy

For immunofluorescence studies, a single well of flexible-bottomed, 6-well plate corresponding to each treatment was first fixed with 4% paraformaldehyde for 10 min at room temperature. Following fixation, a central area from the well was cut out immediately and washed extensively in 1X PBST. The cells were next permeabilized with 0.5% TritonX100 for 10 min, blocked for non-specific binding with 2% BSA and incubated with antiYAP antibody (ab56701, Abcam, Waltham, MA, USA) for 1 h at room temperature. The cells were then incubated with Goat antimouse Alexa Fluor 488 antibody (Invitrogen, Waltham, MA, USA) for 30 min at room temperature. Cytoskeletal staining was accomplished by the addition of Alexa Fluor 594 Phalloidin (Invitrogen) during the secondary antibody incubation. Cells were washed thereafter and mounted onto slides using ProLong Diamond Antifade mount containing DAPI (Invitrogen). Immunofluorescence images were obtained using an inverted fluorescence microscope (Leica).

### 2.9. RNA Extraction and Real-Time Quantitative PCR

RNA extraction was performed using the RNeasy Plus extraction kit (Qiagen, Germantown, MD, USA) following the manufacturer’s instructions. To minimize variability in transcript levels, cells were immediately lysed and processed for RNA extraction following mechanical stress application. Equal amounts of total RNA were used for cDNA generation using the RNA to cDNA EcoDry Premix kit (Takara, San Jose, CA, USA). Real-Time quantitative PCR to access transcript levels was performed in a BioRad CFX96 PCR machine using FAST SYBR Green Master Mix (Applied Biosystems, Waltham, MA, USA) and specific primers for each gene (Table S1).

### 2.10. Statistical Analysis

The difference between observed means for various groups was calculated using an unpaired *t* Test (GraphPad, San Diego, CA, USA). A *p*-value of less than 0.05 (*) was considered as statistically significant with higher levels of significance denoted as ** for *p* < 0.01 and *** for *p* < 0.001.

## 3. Results

### 3.1. Reduced Mineralization-Related Gene Expression, Incomplete Cellular Cementum Mineralization, and 3rd Molar Tooth Row Misalignment in Wnt1cre/YAP Flox/TAZ Flox Mice

**Figure 1:**
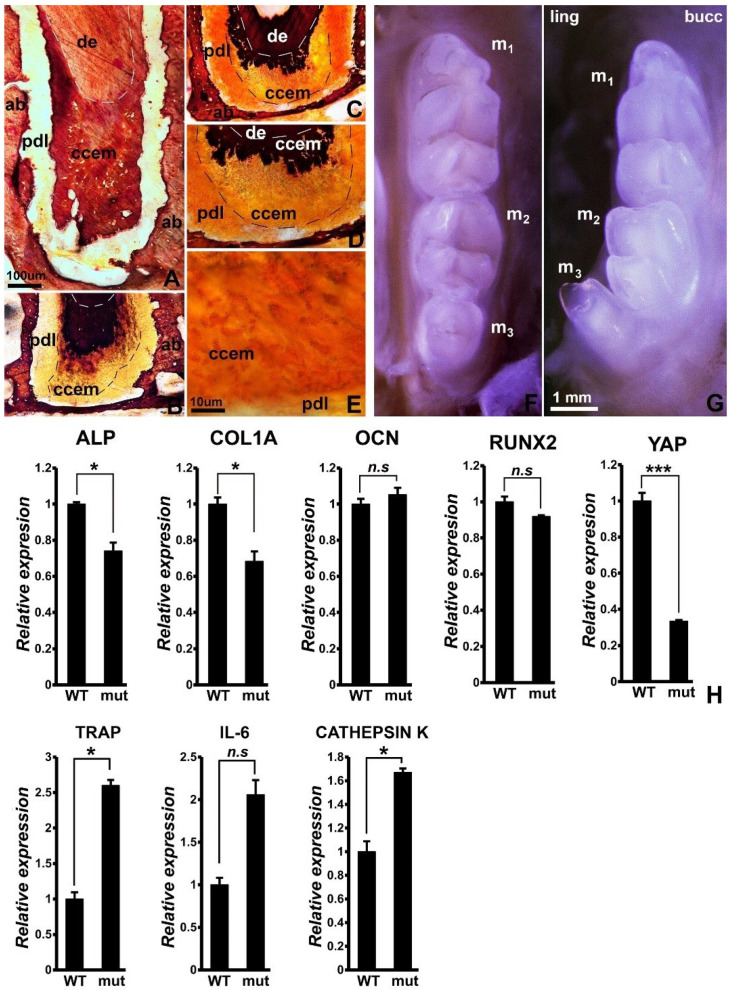
Effect of loss of YAP/TAZ on periodontal homeostasis. (**A**–**E**) Ultrathin ground sections stained with van Kossa’s stain as a mineralization marker. (**A**) is a control and (**B**–**E**) are from mutant mice at increasing levels of magnification. Alveolar bone (ab), root dentin (de), periodontal ligament (pdl), and cellular cementum (ccem) are labeled for orientation purposes. Note the partially mineralized cellular cementum (ccem) in the mutant mice. (**E**) illustrates incomplete mineralization of cellular cementocytes (ccem). (**F**,**G**) Mandibular tooth row comparison between wild type (**F**) and mutant (**G**) mice. The three mandibular molars (m1, m2, m3) are labeled. Note the lingual inclination of the third molar (m3) in YAP1/TAZ mutant mice (**G**). (**H**) Gene expression comparison for select mineralization related markers between wild type and Hippo mutant mice. Representative RT-qPCR for ALP, COL1A, OCN, RUNX2, YAP1, TRAP, IL-6, and cathepsin K transcript levels in mandibles from wild type and Hippo mutants. Transcript levels were normalized to β-actin levels and are presented as fold difference. As expected, YAP1 levels are down 70% in Hippo mutant samples. Representative data from *n* > 3. *t* test for statistical level of significance; *, *p* < 0.05; ***, *p* < 0.005, *n.s*, not significant. The bar in (**A**) is for (**A**–**D**). (**E**) is at a 10× higher magnification.

Loss of YAP/TAZ in Wnt1Cre/YAP/TAZ mice resulted in incomplete cellular cementum mineralization, with the innermost cementum portion displaying solid black van Kossa staining on ultrathin ground sections, while the outer portion revealed only microscopic silver grains in individual cementocytes, compared to the homogeneous staining in the cellular cementum of control mice (Figure 1A–E). On a macroscopic level, tooth rows from Wnt1Cre/YAP/TAZ mice featured a lingually inclined, conical third molar, while molar teeth of wild type controls were oriented in a straight row (Figure 1F,G). To explain these phenotypic changes, the expression levels of key mineralization regulators between Wnt1Cre/YAP/TAZ and wild type mouse mandibles were compared. Transcript levels of YAP1 were significantly reduced by 70% in Wnt1Cre/YAP/TAZ mice when compared to wild type mouse mandibles (Figure 1H). Explaining the hypomineralized alveolar phenotype, the expression of alkaline phosphatase and collagen I mineralization-associated genes was significantly reduced by 25% (ALP) and 30% COL1A) in mandibles from Wnt1Cre/YAP/TAZ mice, while expression of the osteoclast activity markers TRAP and cathepsin K were significantly increased by 2.6-fold (TRAP) and 1.7-fold (cathepsin K). Together, these data demonstrate that Wnt1Cre/YAP/TAZ mice are characterized by a resorptive tilt of the periodontal homeostasis axis and incomplete cellular cementum mineralization.

### 3.2. Cyclic Strain Increased Nuclear YAP, and the Proteoglycan Agrin Partially Rescued the Effect of YAP Knockdown on Nuclear YAP Expression

Our gene expression analysis revealed that the gene expression levels of the extracellular matrix protein collagen (COL1A) were decreased in the periodontium of Wnt1Cre/YAP/TAZ knockout mouse mandibles (Figure 1H). To determine whether COL1A is an essential mediator of Hippo pathway regulation, we subjected periodontal ligament (PDL) cells to cyclical strain mechanical forces. Periodontal ligament cells sustain heavy mechanical forces from biting and chewing and hence are ideal for studying structural and molecular changes accompanying mechanical strain. Cyclic strain was applied on PDL cells grown with or without collagen in combination with siRNA treatment against YAP1 to specifically knockdown YAP1 transcript levels and block the YAP pathway.

**Figure 2:**
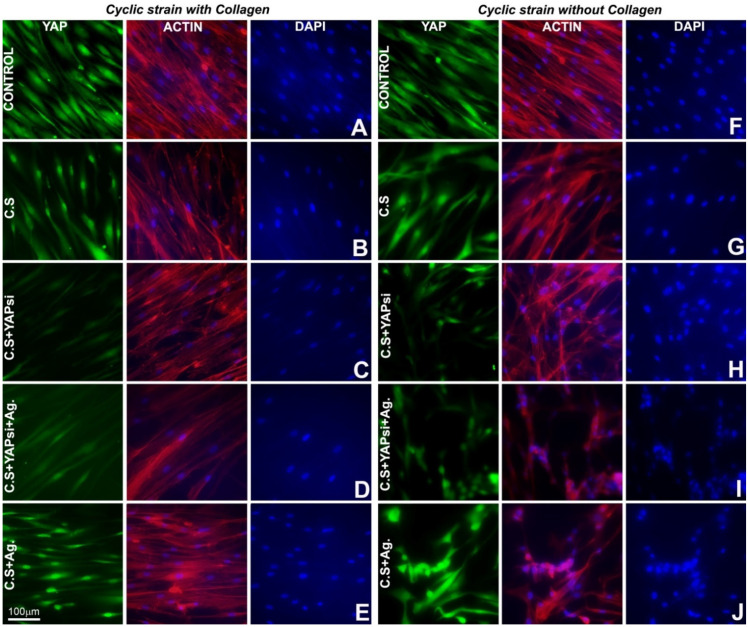
YAP1 localization and cytoskeletal staining during cyclic strain in PDL cells. (**A**–**J**) Representative immunofluorescence images for YAP1 localization and actin distribution following cyclic mechanical strain on PDL cells grown on collagen-coated plates (**B**–**E**) and without collagen (**G**–**J**) under indicated treatment conditions. (**A**,**F**) PDL cells grown with or without collagen and not subjected to any mechanical cyclic strain served as controls. Actin was detected using Alexa Fluor 594 Phalloidin (Red) and is presented in a merged form together with cell nuclei counterstained with DAPI (blue). YAP1 was detected using Alexa Fluor 488 (green). Note the distinctive nuclear localization for YAP1 upon cyclic strain in PDL cells grow on collagen-coated surface (**B**) and to a lesser extent in cells without collagen (**G**).

To assess YAP pathway activation, YAP1 was localized in PDL cells following cyclic strain by immunolocalization, while simultaneously accessing structural and attachment related changes in these cells through F-actin staining. Our studies in control PDL cells revealed a generally diffused localization for YAP1 in both cytoplasm and nucleus while also displaying an elongated fibroblastic shape and extensive staining for F-actin for cells grown with or without collagen (Figure 2A,F). Upon application of cyclic strain, there was a significant level of nuclear translocation for YAP1 in PDL cells grown with collagen compared to control cells without any strain (Figure 2B). On the other hand, YAP1 nuclear translocation after cyclic strain was less pronounced in PDL cells grown without collagen (Figure 2G) when compared to collagen-surface grown PDL cells (Figure 2B). To further understand the role of the YAP pathway as it relates to cyclic strain response, PDL cells were treated with siRNA against YAP1 prior to subjecting them to cyclic strain. YAP1 levels were significantly decreased in siRNA treated cells and the knockdown of YAP1 resulted in extensive cytoskeletal rearrangement and morphological changes in PDL cells grown in the absence of collagen as visualized by F-actin staining (Figure 2H), while these changes were less apparent in PDL cells grown on collagen-coated surfaces (Figure 2C). Addition of the extracellular matrix proteoglycan and known YAP regulator agrin restored the structural integrity of the cytoskeleton in YAP siRNA treated PDL cells grown on collagen-coated plates as demonstrated by F-actin staining (Figure 2D). In contrast, the addition of agrin to PDL cells grown in the absence of collagen with or without YAP siRNA treatment resulted in a drastic change in the cell morphology and cytoskeleton characterized by cell clumping and detachment (Figure 2I,J). Unlike PDL cells grown without collagen, PDL cells grown on collagen-coated plates subjected to cyclic strain and agrin treatment did not exhibit any morphological or cytoskeletal defects and demonstrated normal YAP1 nuclear localization (Figure 2D,E). These studies demonstrated that collagen is essential for physiological YAP1 localization regulation during mechanical strain in PDL cells.

### 3.3. Significant Reduction in Mineralization-Related Gene Expression following YAP1 Knockdown, and Partial Rescue with the Agrin Proteoglycan

**Figure 3:**
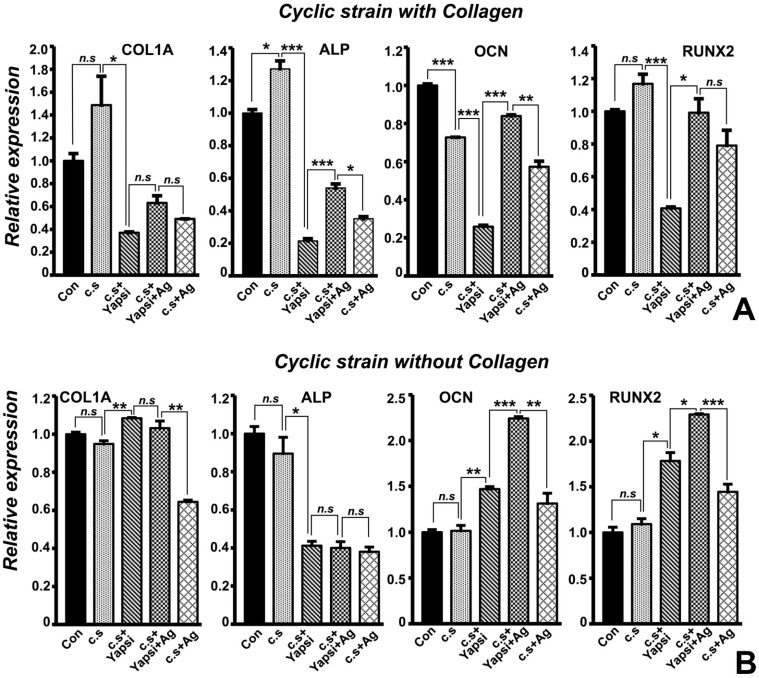
Gene expression analysis for select mineralization and differentiation markers in PDL cells subjected to cyclic strain and controls. Representative RT-qPCR analysis of expression levels for COL1A, ALP, OCN, and RUNX2 in PDL cells grown with collagen (**A**) and without collagen (**B**). Transcript levels for each gene were normalized to β-actin transcript levels. Data are presented as fold difference of expression levels for each experimental treatment condition when compared to cells not subjected to any mechanical strain (Con). Graphs are representative of three independent PCR analysis obtained from at least three experiments for each treatment condition. Addition of agrin rescued ALP, OCN, and RUNX2 transcript levels specifically for PDL cells grown on collagen-coated surfaces (**A**). Con—control; c.s—cyclic strain; Yapsi—YAP1 siRNA treatment; Ag—agrin. *t* test for statistical level of significance; *, *p* < 0.05; **, *p* < 0.01; ***, *p* < 0.005. *n.s*—not significant.

Our immunofluorescence experiments demonstrated a link between collagen and YAP1 during the cyclic strain response in PDL cells. To further probe this link, we investigated gene expression changes associated with mechanical strain response in PDL cells grown with or without collagen and with the addition of YAP siRNA or agrin treatment. Our expression analysis indicated that while intracellular COL1A levels were not significantly altered upon cyclic strain in PDL cells (Figure 3A,B COL1A graph), siRNA mediated knockdown of YAP1 resulted in a significant (>4 fold) decrease in intracellular COL1A transcript levels specifically for PDL cells grown on collagen surfaces (Figure 3A, COL1A graph). Our experiments further revealed that the addition of the proteoglycan agrin did not significantly restore intracellular COL1A transcript levels (Figure 3A, COL1A graph). Surprisingly, COL1A transcript levels were marginally but significantly upregulated in PDL cells grown without collagen when subjected to cyclic strain and YAP1 siRNA treatment (Figure 3B, COL1A graph).

Analysis of ALP early mineralization marker transcript levels revealed a significant 1.3-fold upregulation upon cyclic strain in collagen-surface grown PDL cells, while YAP1 knockdown resulted in a drastic and highly significant 6-fold reduction of ALP transcript levels (Figure 3A, ALP graph). Importantly, addition of agrin restored ALP transcript levels in collagen-grown PDL cells subjected to YAP1 siRNA treatment and cyclic strain (Figure 3A, ALP graph). In comparison, while PDL cells grown without collagen also exhibited a more than 2-fold and significant reduction in ALP transcript levels upon cyclic strain and YAP1 siRNA treatment, this reduction was not rescued with agrin alone (Figure 3B, ALP graph). Furthermore, our study demonstrated that RUNX2 levels were 3-fold downregulated upon YAP1 siRNA treatment and were almost restored completely by agrin in collagen-surface grown PDL cells subjected to cyclic strain (Figure 3A, RUNX2 graph). In contrast to our observations for COL1A, ALP, and RUNX2 transcript levels, OCN transcript levels were significantly downregulated (30% less) after cyclic strain in PDL cells grown with collagen (Figure 3A, OCN graph). YAP1 siRNA treatment further decreased OCN levels more than 2-fold, while agrin completely restored OCN transcript levels in a significant manner (Figure 3A). In tandem with our immunofluorescence localization data, these gene expression results indicate that YAP1 upregulates the expression of key extracellular matrix proteins and mineralization regulators.

### 3.4. Reduced Mineralization-Related Gene Expression and Loss of Tooth/PDL/Alveolar Bone Tissue Contours in the Wnt1Cre/YAP/TAZ Mutant Mice Dentoalveolar Complex following Unloading

**Figure 4:**
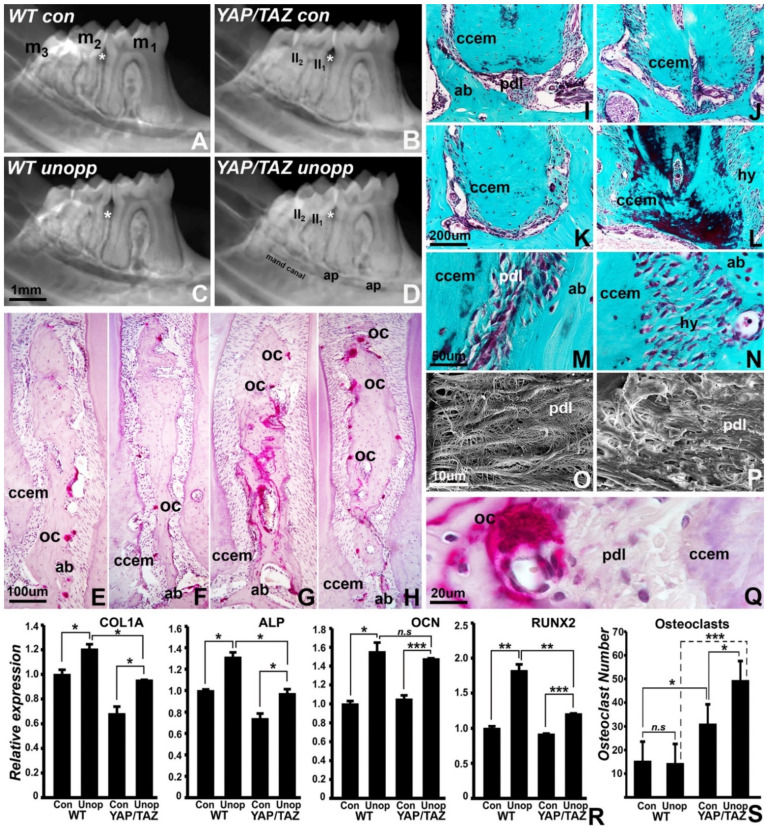
Differential effects of unloading on periodontal microanatomy, homeostasis, and gene expression. (**A**–**D**) X-ray comparison between molar rows from wild type control (**A**), YAP/TAZ control (**B**), wild type subjected to unopposed molar conditions (**C**), and YAP/TAZ subjected to unopposed molar conditions (**D**). Individual molars are labeled as m1, m2, and m3, and tooth roots of the second molar are labeled as II1 and II2. The asterisk indicates the position of the alveolar bone between the first and second molar. Other areas of interest include the root apices of the first molar (ap) and the mandibular canal (mand canal). Note the differences in bone and cementum boundaries. (**E**–**H**) Effect of unloading and of loss of YAP/TAZ on osteoclast activity when comparing wild type control (**E**), YAP/TAZ control (**F**), wild type subjected to unopposed molar conditions (**G**), and YAP/TAZ subjected to unopposed molar conditions (**H**). The alveolar bone wall between the first and second molars was chosen for comparison. The labels demark osteoclasts (oc), cellular cementum (ccem), and alveolar bone (ab). Note the increase in alveolar bone osteoclast activity in the YAP/TAZ mutant mice. (**I**–**L**) Effect of unloading and of loss of YAP/TAZ on second molar first root histology when comparing wild type control (**I**), YAP/TAZ control (**J**), wild type subjected to unopposed molar conditions (**K**), and YAP/TAZ subjected to unopposed molar conditions (**L**). Note the hyalinized periodontal ligament (hy) in between alveolar bone (ab) and cellular cementum (ccem). (**M**–**P**) Comparison between physiological periodontal ligament in wild type (**M**,**O**) and in YAP/TAZ mutant (**N**,**P**) mice using Gomori stained paraffin sections (**M**,**N**) and scanning electron micrographs (**O**,**P**). Note the difference in fiber thickness of the periodontal ligament (pdl) and the hyalinized periodontal ligament (hy) in the mutant mice (**P**). (**Q**) High magnification of one of the alveolar bone osteoclasts at the interface between alveolar bone (ab), periodontal ligament (pdl), and cellular cementum (ccem). (**R**) Gene expression analysis accompanying the unloaded state in wild type and Hippo mutant mouse molars. Transcript levels of COL1A, ALP, OCN, and RUNX2 were normalized to β-actin transcript levels and are presented as fold difference compared to wild type control samples (WT Con). Expression levels were compared between wild type control and wild type unopposed (WT Unop), Hippo control (Hippo Con) and Hippo unopposed (Hippo Unop), and wild type unopposed and Hippo unopposed samples. Both wild type and Hippo mutant samples exhibited increased expression upon unloading, although the extent of upregulation was more in the case of wild type mice compared to Hippo mutants. Data is representative of at least three qRT-PCR analyses of samples from three independent experiments. *t* test for statistical level of significance. (**S**) Difference in osteoclast count between wild type and YAP/TAZ mutant control and unopposed molar dentoalveolar cross-sections. Note the increase in osteoclast numbers in the YAP/TAZ mutant group. *, *p* < 0.05; **, *p* < 0.01; ***, *p* < 0.005. *n.s*—not significant.

To ask the question of whether unloading-related tissue remodeling is uniquely affected by the loss of YAP/TAZ, wild type and Wnt1Cre/YAP/TAZ mice were subjected to unopposed molar model exposure and compared to controls positioned in physiological occlusion. Radiographic comparisons demonstrated that in YAP/TAZ mutant mice, the alveolar bone between the first and second molar had lost periodontal ligament boundaries and contours while the roots of the second molar and the surrounding bone and ligament formed an amorphous electron dense mass without clear separations (Figure 4A–D). There was a similar loss of cementum/bone separations at the apices of the first molar roots (Figure 4A–D). Explaining the diffusion of bone/cementum outlines in mutant mice, there was a substantial increase in osteoclast numbers on paraffin tissue sections, with both the control and the unopposed molar group of the Wnt1Cre/YAP/TAZ mutant group revealing a significant 2-fold and 3-fold increase in osteoclast numbers, respectively (Figure 4E–H,Q,S). Gomori’s connective tissue stain identified areas of periodontal ligament hyalinization and greatly increased periodontal fiber thickness in Wnt1Cre/YAP/TAZ mutant mice (Figure 4I–P). To correlate morphological observations with changes in mineralization-related gene expression, expression levels between control and unopposed mouse mandibles from both wild type and Wnt1Cre/YAP/TAZ mice were compared. There was a significant increase in transcript levels for COL1A, ALP, OCN, and RUNX2 in the unopposed molar model when compared to the corresponding controls for both wild type and Wnt1Cre/YAP/TAZ mice (Figure 4R). ALP and RUNX2 in the unopposed state were upregulated to a lesser degree in the Wnt1Cre/YAP/TAZ group (1.25-fold upregulated for ALP, 1.3-fold upregulated for RUNX2) when compared to the wild type (1.35-fold upregulated for ALP, 1.8-fold upregulated for RUNX2) (Figure 4R, ALP and RUNX2 graphs). Confirming our data from Figure 1, COL1A transcript levels were 0.7-fold downregulated in Wnt1Cre/YAP/TAZ mice, and the unopposed molar state resulted in a significantly elevated expression of COL1A among wild type (1.2-fold) and Wnt1Cre/YAP/TAZ (1.28-fold) mice (Figure 4R). OCN transcript levels were upregulated 1.5-fold upon unloading in Wnt1Cre/YAP/TAZ mice both in control and unopposed molar groups (Figure 4R). In addition, comparison of gene expression levels between wild type and Wnt1Cre/YAP/TAZ unopposed samples revealed a significant level of downregulation for COL1A (1.3-fold), ALP (1.3-fold), and RUNX2 (1.5-fold) transcript levels in Wnt1Cre/YAP/TAZ mice. The reduced expression levels of mineralization-associated genes in Wnt1Cre/YAP/TAZ mice in controls and unloaded conditions further explain the resorptive state of YAP/TAZ mutant mice in unloaded dentoalveolar complexes.

## 4. Discussion

The purpose of the present study was to uncover the role of the Hippo signaling downstream effectors YAP and TAZ on periodontal homeostasis and mechanotransduction. For our studies we employed double-floxed YAP/TAZ mice crossed with Wnt1-Cre as well as YAP knockdown periodontal ligament cells. Successful mouse phenotypes were homozygous for TAZ and heterozygous for YAP when crossed with Wnt1-Cre as per our genotyping results. Mutant mice were studied for changes in mineralization using radiographs and von Kossa reactions as well as gene expression via RT-PCR, while osteoclast activity was monitored using TRAP stain. Periodontal ligament cells were subjected to cyclic strain and analyzed for YAP nuclear and cytoskeletal signals and cytoskeletal configuration using actin. To determine the effect of YAP and TAZ in an unloaded situation, dentoalveolar complex mineralization and mineralization-associated gene expression levels were verified in the unopposed molar model. Together, this study provided a comprehensive analysis of the effects of YAP and TAZ on the mineralization state of the dentoalveolar complex in response to mechanical stress and load.

Our data indicated that loss of YAP/TAZ signaling tilted the periodontal mineral homeostasis toward a catabolic direction: there was a significant reduction in alkaline phosphatase and collagen as well as incomplete mineralization of root cementum in TAZ mutant mice, while the osteoclast markers tartrate-resistant acid phosphatase and cathepsin K as well as osteoclast numbers were significantly upregulated. Moreover, expression levels of mineralization-associated genes collagen, alkaline phosphatase, osteocalcin, and RUNX2 were significantly reduced when YAP was blocked in periodontal ligament progenitors subjected to cyclic strain on collagen-coated dishes. Finally, TAZ mutant mice featured a lingually inclined third molar indicative of a lack of a sufficiently strong alveolus to maintain a straight tooth row under masticatory conditions. Together, these data indicated that YAP is essential for physiological periodontal homeostasis, and loss of YAP/TAZ results in a mineralization defect phenotype. We interpreted the reduction in cementum and bone mineralization and mineralization markers in tandem with the increase in osteoclast activity as evidence of a shift of the periodontal mineralization homeostasis axis into a catabolic direction, suggestive of an uncoupling of osteogenesis and osteoclastogenesis as described in other examples of periodontal tissue loss [26,27,28,29,30]. Several previous studies have suggested that YAP/TAZ signaling promotes mineralization and that lack of YAP/TAZ results in mineralization deficiencies. Specifically, pro-osteogenic effects of YAP on β-catenin signaling [19], effects of Snail/Slug-YAP/TAZ binary complexes on BMSC differentiation [31], a role for YAP on RAMP1-osteogenesis [32], and the effect of YAP on cementoblast differentiation and mineralization [20] were reported. There is no general agreement on the regulatory dynamics of periodontal homeostasis, with some investigators supporting an involvement of pyrophosphate and ANK [33,34], and other investigators favoring Wnt signaling and especially the Wnt antagonist SFRP1 as major factors contributing to the shaping of the unique mineral/soft tissue interface of the periodontium [35,36]. Data presented in this manuscript suggest that the Hippo pathway effectors YAP/TAZ may have a regulatory role in periodontal homeostasis, either by interacting with Wnt signaling or by affecting pyrophosphate balance or by affecting periodontal mineralization-related gene expression directly. The lingually inclined third molar suggests that the YAP/TAZ-induced misregulation of alveolar bone homeostasis affects the third molar periodontium more than the first and second molar. This effect may be due to a thinner bony socket surrounding third molar or to the reduced mineralization level of the third molar socket when compared to the first and second molar sockets.

Our data demonstrated that the ECM proteoglycan agrin rescued the deleterious effect of blocking YAP on mineralized tissue-related gene expression. However, when studies were conducted on uncoated polystyrene dishes devoid of the customary collagen coating, cyclic strain had little effect on gene expression, and blockage of YAP revealed inconsistent effects on mineralization-related gene expression. Previous studies demonstrated that YAP and TAZ function as key mechanotransducers, acting through nuclear relays of mechanical stimuli [37]. In this role, YAP/TAZ are sensors for matrix stiffness, stretch, and cell density, resulting in nuclear localization of YAP upon loading, as verified in the present study. However, the blockage of YAP interfered with its nuclear localization in this study, and this effect was reversed by the addition of agrin to the culture medium, illustrating that even after blocking YAP, increased mechanical stiffness can still induce YAP nuclear localization and increased gene expression. This finding points to the complexities of YAP-dependent mechanotransduction. YAP has been reported to directly involve the Hippo pathway kinases MST and LATS and also function independent of the Hippo pathway [38,39,40]. In addition to being activated by integrin-mediated adhesion, YAP and TAZ are also activated directly by ECM stiffness [41,42]. These studies position YAP in the center of extracellular matrix initiated mechanotransduction and regulation of gene expression. Proper YAP signaling requires physiological ECM stiffness parameters and loads, and changes in load and matrix mechanical properties will affect YAP/TAZ-related gene expression and tissue homeostasis.

On radiographs, boundaries and interfaces between root cementum and alveolar bone were ill-defined in the YAP/TAZ mutant mice, and there was decreased interproximal alveolar bone thickness and root resorption in mutant mice, especially in unloaded teeth. This finding was supported by a significant reduction in COL, ALP, OCN, and RUNX2 mineralization-associated gene expression, especially in the unopposed molar model. Together, these data supported our hypothesis that YAP/TAZ signaling is essential for maintaining periodontal tissue integrity and mineralized tissue contours, especially in unloaded situations. Our study suggests that the reduction in COL, ALP, OCN, and RUNX2 expression levels contributes to the breakdown of alveolar bone/periodontal ligament interfaces as seen on radiographs, but effects on patterning genes or factors such as Wnt genes and their inhibitors might also contribute to the loss of defined boundaries between cementum, periodontal ligament, and alveolar bone. In previous studies we demonstrated that changes in the loading of teeth through hyperocclusion or lack of loading in the unopposed molar model greatly contribute to alveolar bone modeling with increased osteoclast activity [2,43], a finding we confirmed in the present study. However, the reduced levels of mineralization in the YAP/TAZ mutant model due to the reduction in mineralization-related gene expression promoted osteoclast activity in our study and led to a degree of mineralized tissue resorption not seen in the wild type controls.

## Data Availability

Data are available at the Center for Craniofacial Research and Diagnosis, Texas A&M College of Dentistry, upon request.

## Supplementary Materials

The following are available online at https://www.mdpi.com/article/10.3390/jdb10010014/s1, Table S1: PCR primer sequences.
