# Supplementary material for: The Hippo Pathway Effectors YAP/TAZ Are Essential for Mineralized Tissue Homeostasis in the Alveolar Bone/Periodontal Complex
Source: J Dev Biol. 2022 Mar 1;10(1):14. doi: 10.3390/jdb10010014 (PMC8948986; doi:10.3390/jdb10010014)
Supplement: Supplementary file 1 [file jdb-10-00014-s001.zip › jdb-1516079-supplementary.pdf]

Table S1. PCR primer sequences.

Gene	Forward Primer	Reverse Primer
mALP	AACAACCTGACTGACCCTTCG	AATCCTGCCTCCTTCCACC
mOCN	CAGGAGGGCAATAAGGTAGT	CGTAGATAGCGTTTGTAGGC
mRUNX2	TTCTCCAACCCACGAATGCAC	CAGGTACGTGTGGTAGTGAGT
mCOL1A	ACTGGTACATCAGCCCGAAC	TACTCGAACGGGAATCCATC
mYAP1	ACCCTCGTTTTGCCATGAAC	CCTTCTCCATCTGTAACTGC
mACTIN	GCTACAGCTTCACCACCACA	TCTCCAGGGAGGAAGAGGAT
hALP	CAACCCTGGGGAGGAGAC	GCATTGGTGTTGTACGTCTTG
hOCN	GACTGTGACGAGTTGGCTGA	AGCAGAGCGACACCCTAGAC
hRUNX2	CGGAATGCCTCTGCTGTTAT	TGAAACTCTTGCCTCATCCA
hCOL1A	GGAGCTCCAAGGACAAGAAA	ATGAAGGCAAGTTGGGTAGC
hACTIN	TTGCTGACAGGATGCAGAAG	GTACTTGCGCTCAGGAGGAG
